# Role of Fibroblast Growth Factor Receptor 2b in the Cross Talk between Autophagy and Differentiation: Involvement of Jun N-Terminal Protein Kinase Signaling

**DOI:** 10.1128/MCB.00119-18

**Published:** 2018-06-14

**Authors:** Monica Nanni, Danilo Ranieri, Benedetta Rosato, Maria Rosaria Torrisi, Francesca Belleudi

**Affiliations:** a Department of Clinical and Molecular Medicine, Sapienza University of Rome, Laboratory affiliated with Istituto Pasteur Italia-Fondazione Cenci Bolognetti, Rome, Italy; b Sant'Andrea University Hospital, Rome, Italy

**Keywords:** FGFR2b, JNK, autophagy, differentiation, keratinocyte

## Abstract

Fibroblast growth factor receptor 2b (FGFR2b) is a receptor tyrosine kinase expressed exclusively in epithelial cells. We previously demonstrated that FGFR2b induces autophagy and that this process is required for the triggering of FGFR2b-mediated early differentiation of keratinocytes. However, the molecular mechanisms regulating this interplay remain to be elucidated. Since we have also recently shown that Jun N-terminal protein kinase 1 (JNK1) signaling is involved in FGFR2b-induced autophagy and a possible role of the JNK pathway in epidermal differentiation has been suggested (though it is still debated), we investigated here the cross talk between FGFR2b-mediated autophagy and differentiation, focusing on the downstream JNK signaling. Biochemical, molecular, and immunofluorescence approaches in 2-dimensional (2-D) keratinocyte cultures and three-dimensional (3-D) organotypic skin equivalents confirmed that FGFR2b overexpression increased both autophagy and early differentiation. The use of FGFR2b substrate inhibitors and the silencing of JNK1 highlighted that this signaling is required not only for autophagy but also for the triggering of early differentiation. In contrast, the extracellular signal-regulated kinase 1 and 2 (ERK1/2) pathway did not appear to be involved in the two processes, and AKT signaling, whose activation contributes to the FGFR2b-mediated onset of keratinocyte differentiation, was not required for the triggering of autophagy. Overall, our results point to JNK1 as a signaling hub that regulates the interplay between FGFR2b-induced autophagy and differentiation.

## INTRODUCTION

The epithelial isoform of fibroblast growth factor receptor 2/keratinocyte growth factor receptor (FGFR2b/KGFR) is a well-recognized regulator of keratinocyte differentiation and skin homeostasis (1–3). In agreement with this role, studies from our group have demonstrated that FGFR2b has a key function in the regulation of epidermal early differentiation (4–6) and that the downstream phosphoinositide 3-kinase (PI3K/AKT) signaling is involved in this process (4).

Recently, a close interplay between differentiation and autophagy has been proposed for many cell types, including keratinocytes (7–11). Although most of the evidence suggests a crucial role for the autophagic machinery during the progression toward terminal differentiation of keratinocytes (7–9), other findings indicate its possible positive role even in the early stages of the process (7, 9–11). In fact, some autophagic markers have been detected starting from the basal/suprabasal layers of the epidermis (7, 9), where they increase in parallel with the early differentiation markers keratin 1 (K1) and keratin 10 (K10) (7, 10, 11), and K1 expression can be repressed by the inhibition of autophagy (10, 11). Overall, these observations have suggested the existence of positive cross talk between autophagy and cell differentiation.

Previous studies from our group have shown that the FGFR2b-specific ligand FGF7/KGF triggers autophagy in keratinocytes (10) and that this effect depends on FGFR2b expression and activation through a PI3K/AKT/mTOR-independent pathway (10, 12). In addition, we also demonstrated that the autophagic process is required for receptor-mediated early differentiation (10), suggesting that, at least in keratinocytes, autophagy and differentiation could be interplaying processes regulated by FGFR2b. Therefore, we decided to identify here the receptor downstream signaling pathways and molecular players regulating this cross talk. To this aim, we focused our attention on the Jun N-terminal protein kinase (JNK) family, a group of well-known signaling pathway-activated downstream tyrosine kinase receptors, including FGFRs (13). JNK1 activation is involved in MTOR-independent autophagy (14) and has recently been described as a crucial mediator in FGFR4-induced autophagy in chondrocytes (15). Accordingly, recent findings of our group have shown that the JNK1 pathway, activated via the upstream substrate phospholipase C-gamma (PLC-γ), is also implicated in the autophagy triggered by FGFR2b in keratinocytes (16). Interestingly, JNK has, on the other hand, also been proposed as a regulator of keratinocyte differentiation, even if its role still remains unclear and widely controversial; in fact, while microarray approaches have indicated a repressive role in keratinocyte early and late differentiation (17), other published results demonstrated that JNK is required for K1 expression (18), which is controlled by the JNK downstream transcription factor Jun/AP-1 (19). Therefore, JNK could be required to allow the onset of early keratinocyte differentiation triggered by FGFR2b and may represent a signaling hub at the crossroad between autophagy and epidermal differentiation.

## RESULTS AND DISCUSSION

### FGFR2b expression and signaling trigger both autophagy and early differentiation during the keratinocyte shift from basal to suprabasal layers.

**FIG 1:**
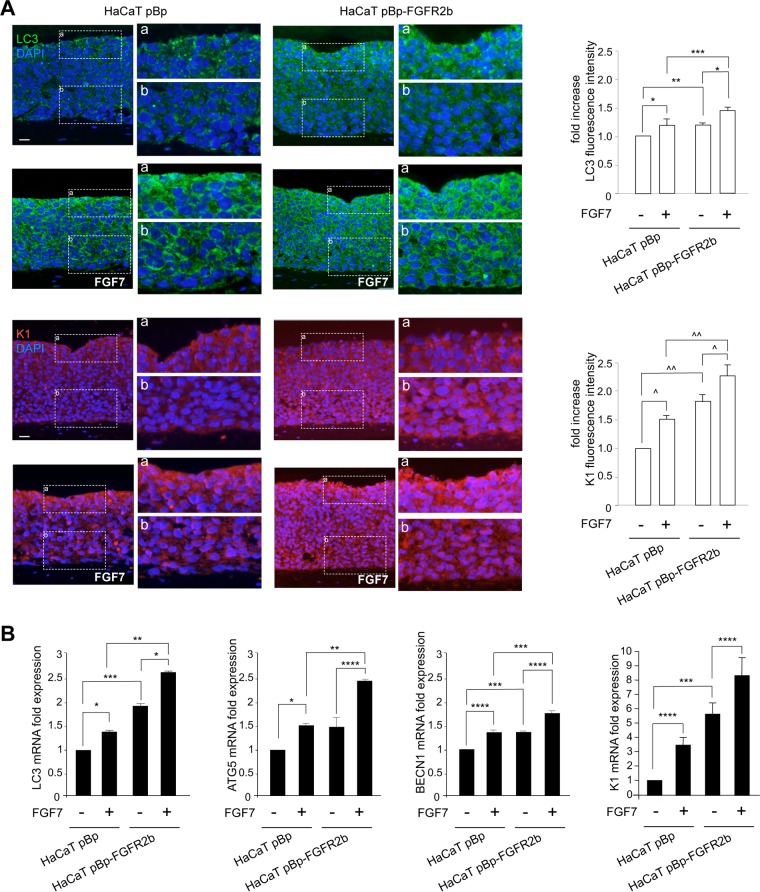
FGFR2b expression and signaling trigger both autophagy and epidermal early differentiation. Organotypic skin equivalents using HaCaT pBp and pBp-FGFR2b clones, prepared as reported in Materials and Methods, were grown in complete medium and then left untreated or stimulated with FGF7 for an additional 4 days. (A) Quantitative immunofluorescence analysis shows that, in pBp rafts stimulated with FGF7, LC3 and K1 staining appear intense starting from the first suprabasal layer. In HaCaT pBp-FGFR2b rafts, both stains are already detectable in the basal layer and are increased compared to the levels in controls, particularly after FGF7 stimulation. Quantitative analysis of the fluorescence intensities was performed as described in Materials and Methods, and the results are expressed as fold increases with respect to mean values ± standard errors (SE) for pBp cells. Student's *t* test was performed, and significance levels are defined as *P* values of <0.05. For comparison to the results for the corresponding FGF7-unstimulated cells: *, *P* < 0.01; ^, *P* < 0.001. For comparison to the results for the corresponding pBp cells: **, *P* < 0.0001; ***, *P* < 0.05; ^^, *P* < 0.001. Bar = 25 μm. (B) Real-time RT-PCR analysis shows that mRNA expression levels of LC3, ATG5, and BECN1, as well as of K1, are significantly increased upon FGF7 stimulation, particularly in pBp-FGFR2b rafts. Results are expressed as mean values ± SE. Student's *t* test was performed, and significance levels are defined as *P* values of <0.05. For comparison to the results for the corresponding FGF7-unstimulated cells: *, *P* < 0.01; ****, *P* < 0.05. For comparison to the results for the corresponding pBp cells: **, *P* < 0.01; ***, *P* < 0.05.

To analyze in parallel the FGFR2b-induced autophagy and differentiation in human keratinocytes, we first investigated the expression and distribution throughout the epidermal layers of both the autophagic marker LC3 and the early differentiation marker K1. To this aim, we reproduced *in vitro* the keratinocyte differentiation steps that occur *in vivo*, using three-dimensional (3-D) organotypic skin equivalents engineered with human HaCaT keratinocytes (20) stably transduced with pBp-FGFR2b retroviral constructs or with empty pBp vector as a negative control (21). The organotypic cultures were left untreated or stimulated with FGF7. Quantitative immunofluorescence analysis demonstrated that, in unstimulated pBp rafts, the dotted staining of LC3 was mainly visible in the uppermost layers (Fig. 1A), where it is well known that autophagy (7–9) and nucleophagy (9) are mostly active for terminal differentiation. In the corresponding FGF7-stimulated pBp rafts, the LC3 signal appeared more intense and already visible starting from the first suprabasal layer (Fig. 1A). Parallel evaluation of K1 expression showed that, in pBp rafts, the signal of this differentiation marker was detectable in all suprabasal layers, as expected (6, 22). In addition, in agreement with the well established role of FGFR2b and its signaling in keratinocyte differentiation (4–6), K1 staining was increased by FGF7 stimulation (Fig. 1A). The expression of both autophagic and differentiation markers appeared clearly enhanced by FGFR2b overexpression, as expected (4, 6, 10, 12, 16): in fact, in HaCaT pBp-FGFR2b rafts, the LC3 and K1 signals were overall increased compared to the results for controls, particularly after FGF7 stimulation (Fig. 1A). In addition, both the autophagic and differentiation markers were clearly visible starting already from the basal layer (Fig. 1A), confirming that the forced expression of FGFR2b is able to induce not only the precocious onset of early differentiation but also a simultaneous precocious induction of autophagy. To further validate our results at the molecular level, we then analyzed the mRNA expression levels of LC3, of other key autophagic genes, such as the ATG5 and BECN1 genes, and of K1. All the genes considered appeared to have strongly and significantly increased expression at the transcript level in response to FGF7, particularly in pBp-FGFR2b rafts (Fig. 1B).

Thus, the interplay between autophagy and differentiation triggered by FGFR2b appears to take place at the shift of keratinocytes from the basal to the suprabasal layers.

### JNK1 is a signaling hub that regulates FGFR2b-induced autophagy and differentiation in keratinocytes.

To search for the FGFR2b downstream signaling pathway linking autophagy and differentiation, HaCaT clones were grown just until confluence, the step that *in vivo* precedes the shift from basal to suprabasal layers, where the interplay between the two processes occurs. Confluent cultures were left untreated or stimulated with FGF7. Western blot analysis confirmed that the levels of both the 16-kDa autophagosomal membrane-associated LC3 form, LC3-II, and the early differentiation marker K1 appeared clearly upregulated by FGF7 stimulation, particularly in cells overexpressing FGFR2b (see Fig. S1A, left and central panels, in the supplemental material). Similar results were obtained for desmoglein-1 (DSG1) (Fig. S1A, central panel), a desmosomal component directly involved in the initiation of early differentiation (23, 24). In contrast, an opposite behavior was observed for β1-integrin, a marker for basal undifferentiated keratinocytes whose expression is lost during the onset of differentiation (25): in fact, the expression of this adhesion molecule appeared unaltered in HaCaT pBp cells (Fig. S1A) but decreased in HaCaT pBp-FGFR2b cells, particularly in response to FGF7 stimulation (Fig. S1A). Finally, no changes in E-cadherin expression were observed in either clone (Fig. S1A), consistent with the role of this adhesion molecule as a constituent of the adherent junctions that is widely expressed throughout all the epidermal layers (22). Quantitative immunofluorescence approaches highlighted that the intensity of the LC3 signal, as well as that of K1 staining, was enhanced by FGF7 stimulation, particularly in cells overexpressing FGFR2b (Fig. S1B), while the β1-integrin signal appeared strongly reduced and delocalized from the plasma membrane (Fig. S1B), suggesting the internalization and possible degradation of this marker. Molecular approaches showed that, in 2-dimensional (2-D) cultures, the changes of the expression signatures of some key autophagic genes (LC3, ATG5, and BECN1 genes) and early differentiation genes (K10 and DSG1 genes) in response to FGFR2b overexpression and/or FGF7 stimulation (Fig. S1C) were comparable to those observed in the corresponding skin equivalents (Fig. 1B). The overexpression of FGFR2b in HaCaT pBp-FGFR2b cells compared to its expression in control cells was verified at the protein and mRNA transcript levels using biochemical (Fig. S1A), immunofluorescence (Fig. S1B), and molecular approaches (Fig. S1C). Thus, confluent cultures, which efficiently mimic the shift from undifferentiated to differentiating keratinocytes, are a suitable approach to investigate the signaling pathways downstream from FGFR2b involved in the regulation of the cross talk between receptor-induced autophagy and differentiation.

**FIG 2:**
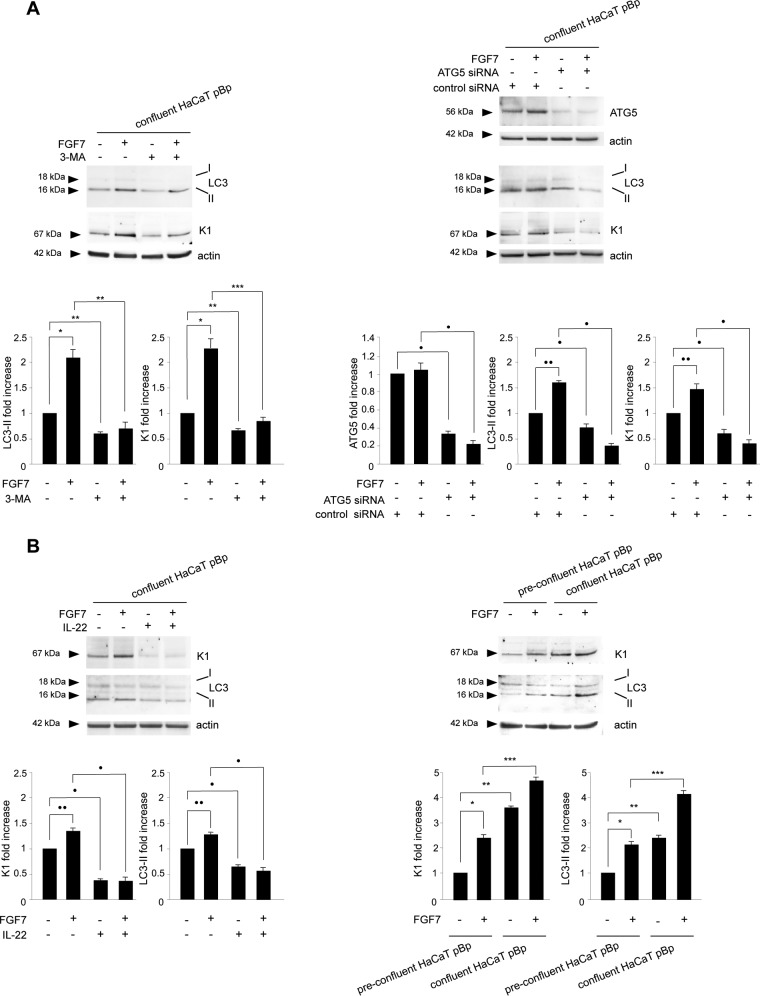
FGFR2b-triggered autophagy and differentiation are linked by a positive loop. (A) HaCaT pBp clones were grown until confluence and left untreated or stimulated with FGF7 in the presence or absence of 3-MA (left). Alternatively, cells were transfected with a small interfering RNA for ATG5 or with an unrelated siRNA as a control before FGF7 stimulation (right). Western blot analysis shows that both the presence of 3-MA (left) and the ATG5 siRNA transfection (right) reduced the levels of both basal and FGF7-induced LC3-II and K1. Equal loading was assessed with antiactin antibody. For the densitometric analysis, the values from 3 independent experiments were normalized and expressed as fold increases and are reported as mean values ± standard deviations (SD). Student's *t* test was performed, and significance levels are defined as *P* values of <0.05. *, *P* < 0.05 versus the results for the corresponding FGF7-unstimulated cells; **, *P* < 0.05 versus the results for the corresponding 3-MA-untreated cells; ***, *P* < 0.01 versus the results for the corresponding 3-MA-untreated cells; ·, *P* < 0.05 versus the results for the corresponding control siRNA cells; ··, *P* < 0.05 versus the results for the corresponding FGF7-unstimulated cells. (B) HaCaT pBp clones were left untreated or stimulated with FGF7 in the presence or absence of IL-22 as reported in Materials and Methods (left). Alternatively, cells were grown until reaching different levels of confluence before FGF7 stimulation (right). Western blot analysis shows that the presence of IL-22 (left), as well as the preconfluence condition (right), reduces the expression levels of K1 and LC3-II, particularly in cells stimulated with FGF7. Equal loading was assessed with antiactin antibody. Densitometric analysis and Student's *t* test were performed as reported above. ·, *P* < 0.05 versus the results for the corresponding IL-22-untreated cells; ··, *P* < 0.05 versus the results for the corresponding FGF7-unstimulated cells; *, *P* < 0.05 versus the results for the corresponding FGF7-unstimulated cells; **, *P* < 0.05 versus the results for the corresponding preconfluent cells; ***, *P* < 0.01 versus the results for the corresponding preconfluent cells.

In order to first assess the relationship between FGFR2b-controlled autophagy and differentiation, we interfered alternatively with the two processes and looked at the effects of each block. In agreement with our previous data (10), Western blot analysis demonstrated that the blocking of autophagy by the general inhibitor 3-methyladenine (3-MA) (26–28) was able to reduce not only the increase in LC3-II in response to FGF7 (Fig. 2A, left) but also that of the K1 marker (Fig. 2A, left). Comparable results for FGF7-dependent induction of both the LC3-II and K1 marker were also obtained by inhibiting the autophagic process by efficient depletion of ATG5 through ATG5-specific small interfering RNA (siRNA) transfection (Fig. 2A, right). In fact, ATG5 is a crucial player in early stages of autophagosome formation (29), whose depletion also specifically blocks autophagy in keratinocytes (10, 30). Smaller effects of both 3-MA treatment and ATG5 depletion were also evident in cells not stimulated with FGF7 (Fig. 2A, left and right), confirming that the autophagic process is also required for the spontaneous onset of differentiation (11). On the other hand, the inhibition of cell differentiation obtained by treatment with interleukin-22 (IL-22) (Fig. 2B, left), a well-established inhibitor of keratinocyte differentiation (31–33), was able to downregulate not only the basal and FGF7-induced expression of K1 but also that of LC3-II. Similar results were obtained in HaCaT cultures in which the differentiation program was inhibited, maintaining cells in a preconfluent condition (Fig. 2B, right). Overall, these lines of evidence indicated that FGFR2b-controlled autophagy and differentiation are linked by a positive loop. These lines of evidence are consistent with previous reports describing the existence of a positive interplay between basal autophagy and spontaneous differentiation in keratinocytes (7–11).

**FIG 3:**
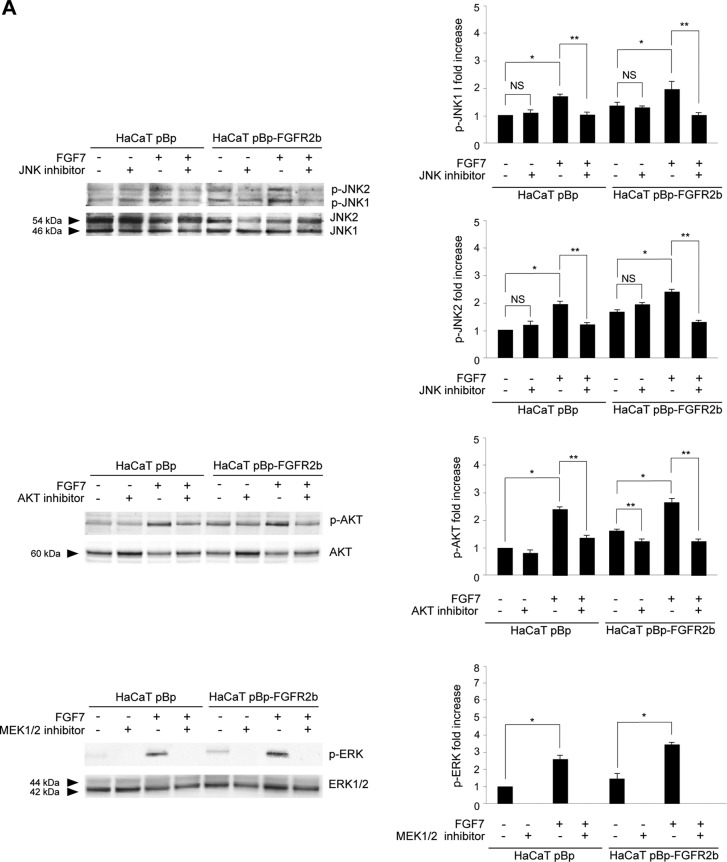

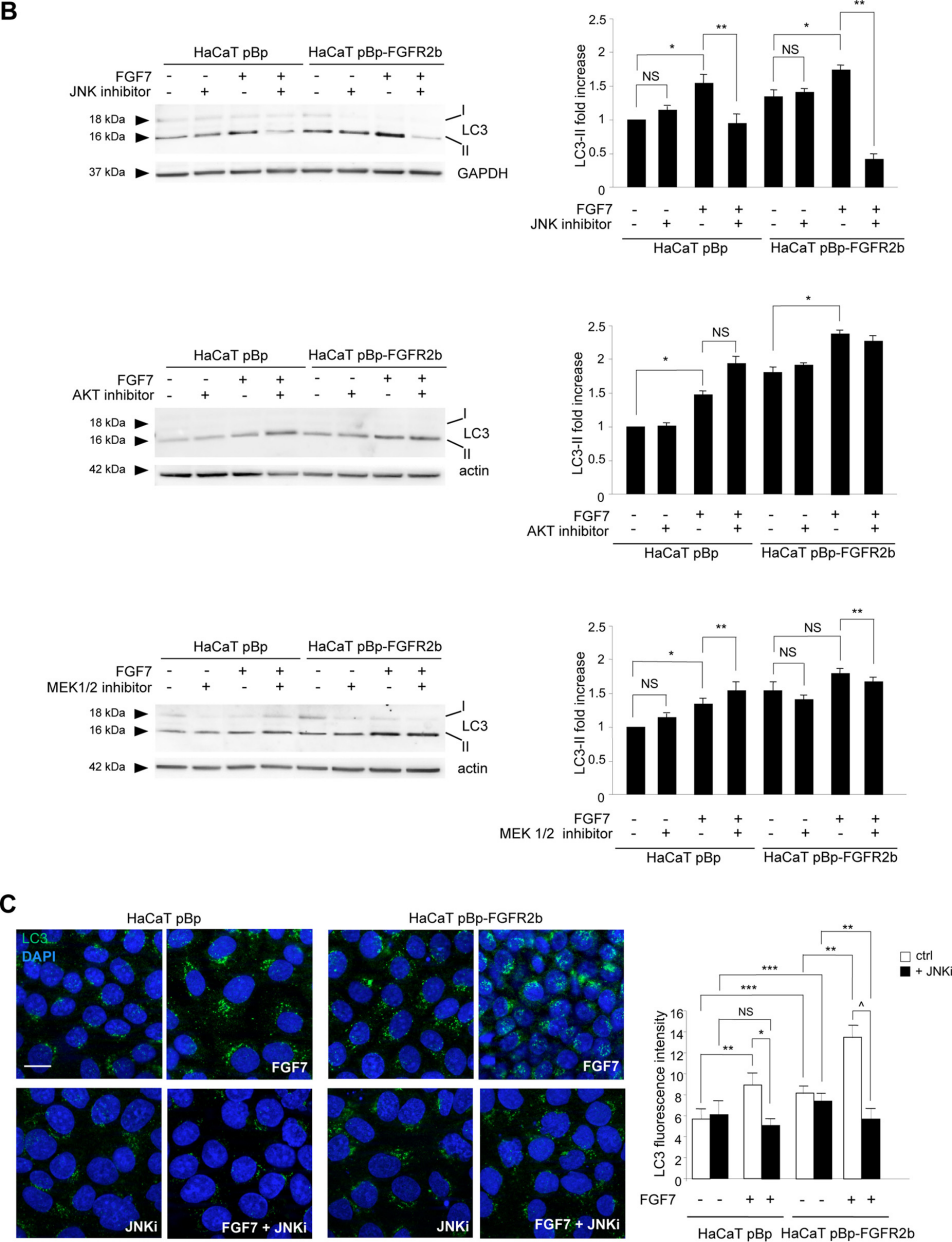
JNK signaling is required for FGFR2b-induced autophagy. (A, B) HaCaT pBp and HaCaT pBp-FGFR2b clones were left untreated or stimulated with FGF7 in the presence or absence of the indicated substrate inhibitors as reported in Materials and Methods. (A) Western blot analysis performed using antibody directed against the phosphorylated form of each substrate confirms the efficiency of all the inhibitors. Equal loading was assessed with anti-JNK1/2, anti-AKT, and anti-ERK1/2 antibodies. Densitometric analysis and Student's *t* test were performed as reported in the legend to Fig. 2. *, *P* < 0.05 versus the results for the corresponding FGF7-unstimulated cells. For comparison to the results for the corresponding substrate inhibitor-untreated cells: **, *P* < 0.05; NS, not significant. (B) Western blot analysis shows that only the JNK inhibitor, and not the AKT or MEK1/2 inhibitor, impairs the increase in LC3-II levels induced by FGF7. Equal loading was assessed with antiactin and anti-GAPDH antibodies. Densitometric analysis and Student's *t* test were performed as reported in the legend to Fig. 2. *, *P* < 0.05 versus the results for the corresponding FGF7-unstimulated cells. For comparison to the results for the corresponding substrate inhibitor-untreated cells: **, *P* < 0.05; NS, not significant. (C) Quantitative immunofluorescence analysis shows that in the HaCaT pBp clone and even more in the HaCaT pBp-FGFR2b clone, the JNK inhibitor is able to interfere with the increase in LC3 signal intensity induced by FGF7. Quantitative analysis and Student's *t* test were performed as reported in the legend to Fig. 1A. For comparison to the results for the corresponding control cells: *, *P* < 0.001; ^, *P* < 0.0001. For comparison to the results for the corresponding FGF7-unstimulated cells: **, *P* < 0.001; NS, not significant. ***, *P* < 0.001 versus the results for the corresponding pBp cells. Bar = 10 μm.

**FIG 4:**
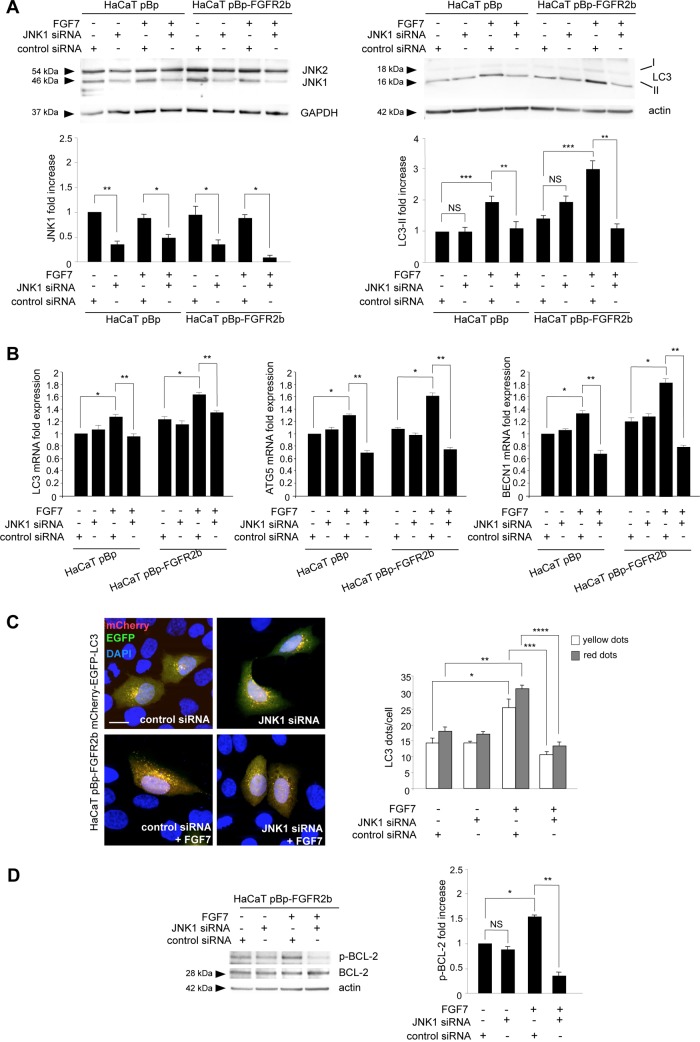
JNK1 plays a role in both FGFR2b-mediated transcriptional control and posttranslational control of autophagy. (A, B) HaCaT pBp and HaCaT pBp-FGFR2b clones were transiently transfected with JNK1 siRNA or an unrelated siRNA as a control. Cells were then left untreated or stimulated with FGF7 as described in Materials and Methods. (A) Western blot analysis using anti-JNK1/2 antibody shows that JNK1 siRNA induces efficient depletion of JNK1 (left). JNK1 silencing strongly affects the FGF7-induced increase in LC3-II (right). Equal loading was assessed with antiactin and anti-GAPDH antibodies. Densitometric analysis and Student's *t* test were performed as reported in the legend to Fig. 2. For comparison to the results for the corresponding control siRNA cells: *, *P* < 0.05; **, *P* < 0.01; NS, not significant. ***, *P* < 0.05 versus the results for the corresponding FGF7-unstimulated cells. (B) Real-time RT-PCR analysis shows that the increases in LC3, ATG5, and BECN1 mRNA transcripts induced by FGF7 stimulation are significantly counteracted by JNK1 depletion. Results are expressed as mean values ± SE. Quantitative analysis and Student's *t* test were performed as as reported in the legend to Fig. 1B. *, *P* < 0.05 versus the results for the corresponding FGF7-unstimulated cells; **, *P* < 0.05 versus the results for the corresponding control siRNA cells. (C) HaCaT pBp-FGFR2b cells were cotransfected with JNK1 siRNA or control siRNA and mCherry-EGFP-LC3. Cells were then left untreated or stimulated with FGF7 as described in Materials and Methods. Quantitative fluorescence analysis shows that JNK1 depletion significantly inhibits the increases in both yellow and red dots per cell induced by FGF7 stimulation. Quantitative analysis was performed as described in Materials and Methods, and results are expressed as mean values ± SE. Student's *t* test was performed as reported in the legend to Fig. 1A. For comparison to the results for the corresponding FGF7-unstimulated cells: *, *P* < 0.05; **, *P* < 0.001. For comparison to the results for the corresponding control siRNA cells: ***, *P* < 0.05; ****, *P* < 0.01. Bar = 10 μm. (D) HaCaT pBp-FGFR2b clones transfected with JNK1 siRNA or control siRNA were left untreated or stimulated with FGF7 as described in Materials and Methods. Western blot analysis shows that the increase in BCL-2 phosphorylation induced by FGF7 stimulation is significantly repressed by JNK1 depletion. Equal loading was assessed with anti-BCL-2 antibody. Densitometric analysis and Student's *t* test were performed as reported in the legend to Fig. 2. *, *P* < 0.05 versus the results for the corresponding FGF7-unstimulated cells. For comparison to the results for the corresponding control siRNA cells: **, *P* < 0.01; NS, not significant.

Then, searching for the possible downstream candidates that could control the cross talk between FGFR2b-triggered autophagy and receptor-mediated differentiation, we focused our attention on JNK because of its involvement in FGFR-triggered autophagy (15, 16), as well as in keratinocyte differentiation (17, 18). In parallel, we also considered the PI3K/AKT signaling pathway, which we have previously demonstrated to play a role in FGFR2b-induced keratinocyte differentiation (4) but not in receptor-mediated autophagy (10), and the extracellular signal-regulated kinase 1 and 2 (ERK1/2) pathway, which we have recently found not to be involved in FGFR2b-induced autophagy (16). Impairment of the three pathways was obtained using a JNK-specific inhibitor, SP600125 (15, 16), an AKT-specific inhibitor, AKT-I-1/2 (34), or an inhibitor of the ERK1/2 upstream substrates MEK1/2, PD0325901 (16, 35), whose efficiency was first confirmed by Western blotting (Fig. 3A). In a second step, we confirmed the effects of each inhibitor on LC3-II expression in confluent HaCaT pBp and HaCaT pBp-FGFR2b clones left untreated or stimulated with FGF7. As expected (10, 16), Western blot analysis showed that the JNK inhibitor, but not the AKT and MEK1/2 inhibitors, clearly impaired the increases in LC3-II levels induced by FGF7 stimulation in pBp and, more evidently, in pBp-FGFR2b cells (Fig. 3B). Interestingly, no evident effects of the JNK inhibitor were detectable in cells not stimulated with FGF7, suggesting that, at least in our keratinocyte model, JNK signaling shutdown is able to efficiently block FGF7-induced but not basal autophagy. The involvement of the JNK pathway in FGFR2b-mediated autophagy was also confirmed by immunofluorescence approaches, demonstrating that in both cell clones, the JNK inhibitor was able to interfere with the increase in the LC3 signal induced by FGF7 (Fig. 3C). In order to unequivocally demonstrate that the effects of the JNK inhibitor on LC3-II levels were attributable to the inhibition of JNK1, the main JNK family member involved in the induction of autophagy (14), we performed specific protein depletion by siRNA transfection. To this aim, HaCaT clones were transfected with JNK1 siRNA or with an unrelated siRNA as a control. The efficiency of JNK1 depletion was verified through Western blot analysis (Fig. 4A, left). After siRNA transfection, cells were grown until confluence and left untreated or stimulated with FGF7 as described above. Western blot analysis showed that JNK1 silencing strongly affects the FGF7-induced increase in LC3-II but not its basal level (Fig. 4A, right), indicating the specific involvement of JNK1 signaling in FGF7-triggered autophagy.

It has recently been demonstrated that JNK1 can induce autophagy via the upregulation of several ATG genes, such as the LC3, ATG5, and BECN1 genes (36, 37), and our previous findings showed that these genes are significantly induced by FGF7 stimulation (12). Molecular approaches performed in HaCaT clones, transfected with JNK1 siRNA or control siRNA and treated with FGF7 as described above, clearly showed that the up-modulation of these genes induced by FGF7 was significantly inhibited by JNK1 depletion (Fig. 4B). Thus, the transcriptional control of FGFR2b on autophagy is mediated by its downstream JNK1 pathway.

Since we have previously demonstrated that FGFR2b signaling is able to stimulate both the formation of autophagosomes and their turnover (10), we wondered in which of the two events JNK1 signaling is implicated. To answer this question, fluorescence experiments were performed by cotransfecting HaCaT pBp-FGFR2b cells with JNK1 siRNA or control siRNA and the pDest-mCherry-EGFP-LC3 autophagic flux sensor (38). Since enhanced green fluorescent protein (EGFP) fluorescence is quenched in acidic environments, while mCherry is an acid-stable fluorescent tag, the newly forming autophagosomes will display both red and green (yellow) fluorescent labels, whereas the acidic autolysosomes only display red fluorescence, as a consequence of the EGFP quenching. Transfected cells were left untreated or stimulated with FGF7 as described above. Quantitative fluorescence analysis showed that JNK1 depletion significantly inhibited the FGF7-induced increase in both yellow and red dots (Fig. 4C), indicating that JNK1 signaling is implicated in the FGFR2b-triggered assembly of new autophagosomes. Again, the absence of detectable effects of JNK1 depletion on the number of yellow/red dots in cells not stimulated with FGF7 confirmed that JNK1 signaling does not affect basal autophagy.

It is well known that JNK1 signaling triggers mTOR-independent autophagy via BCL-2 phosphorylation and consequent beclin-1 release from the BCL-2/beclin-1 inhibitory complex (14, 36, 39). Released beclin-1 can be recruited to the class III PI3K complex, allowing the very early step of phagophore nucleation (40). This JNK1-dependent mechanism has been previously described in starvation-induced (39) and in FGFR4-triggered autophagy (15). In order to assess whether this mechanism could also underlie FGFR2b-triggered autophagy, we checked the BCL-2 phosphorylation levels in HaCaT pBp-FGFR2b cells alternatively transfected with JNK1 siRNA or control siRNA and stimulated or not stimulated with FGF7 as described above. The results clearly showed that FGF7 stimulation increased BCL-2 phosphorylation, which was significantly repressed by JNK1 depletion (Fig. 4D). These results suggest that FGFR2b downstream signaling mediated by JNK1 appears to specifically act at the level of the phagophore nucleation.

**FIG 5:**
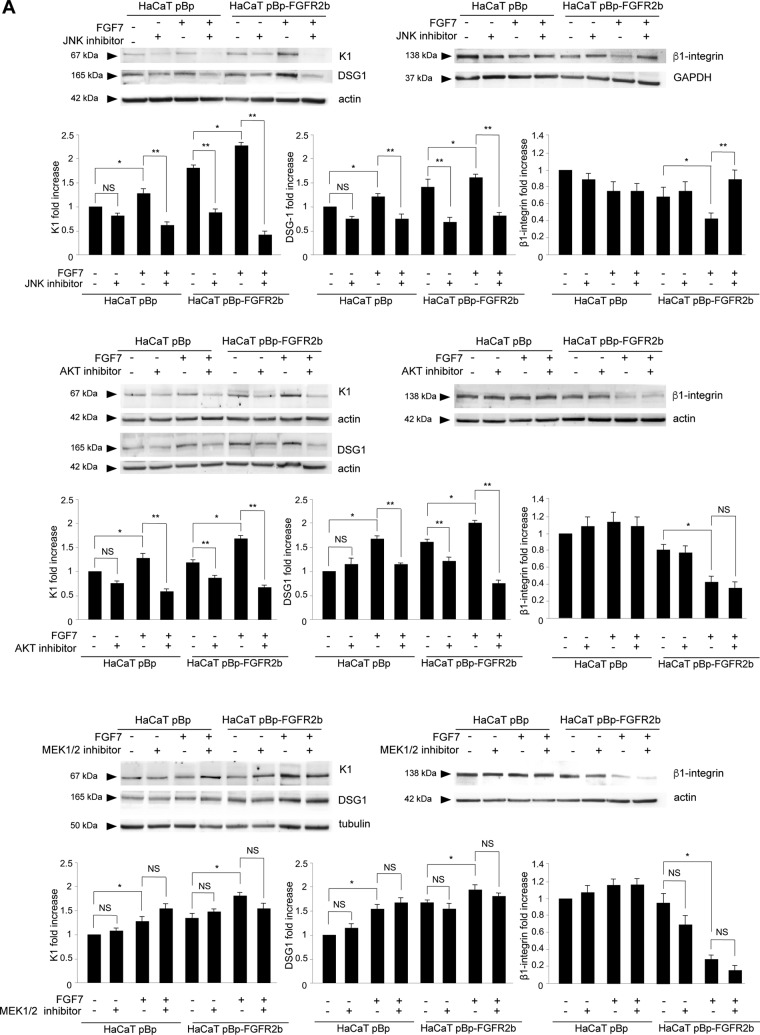

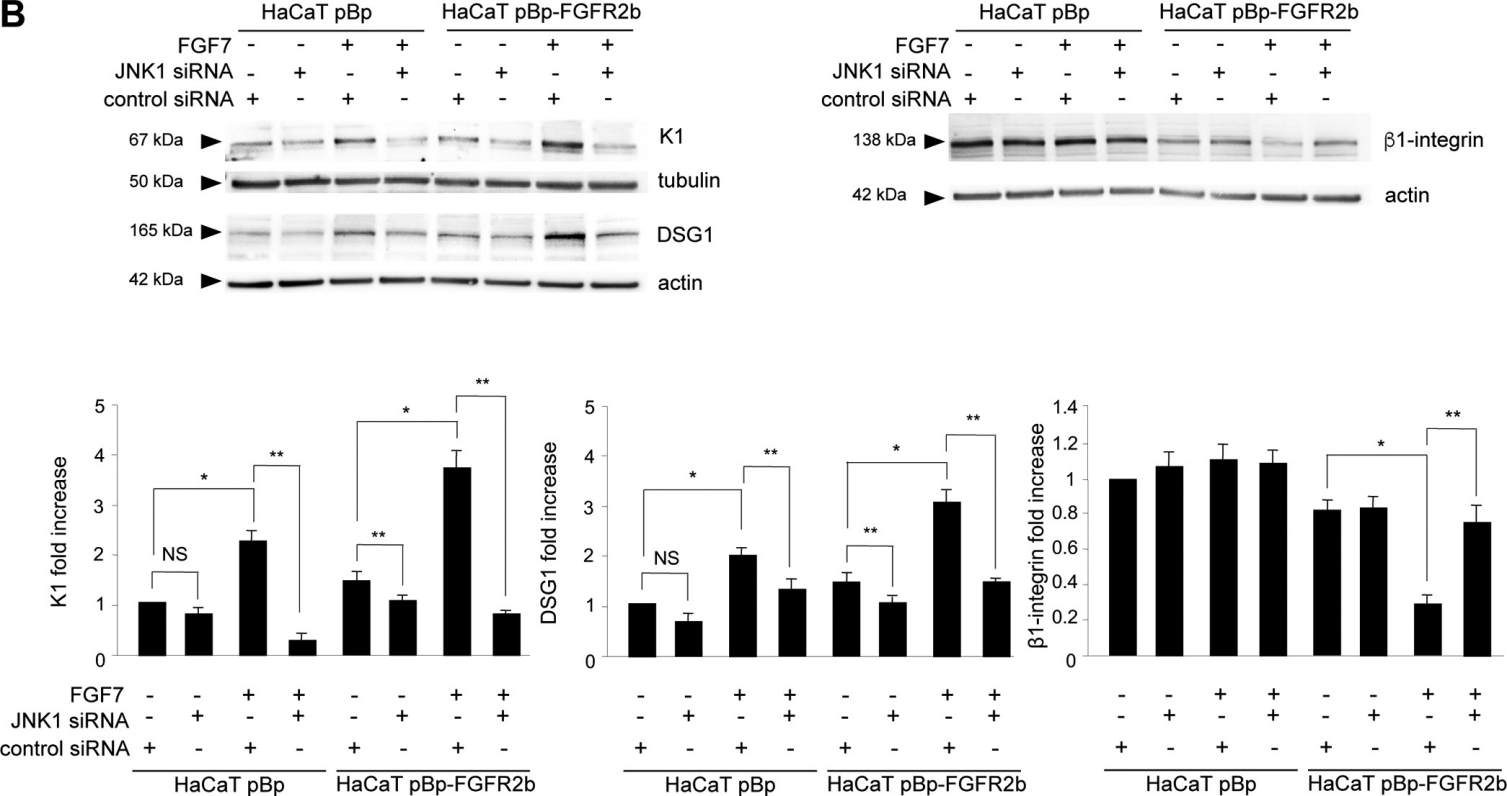
JNK1 signaling is involved in FGFR2b-triggered early differentiation. (A) HaCaT pBp and HaCaT pBp-FGFR2b cells were left untreated or stimulated with FGF7 in the presence or absence of the indicated substrate inhibitors as described in Materials and Methods. Western blot analysis shows that the JNK inhibitor strongly impairs the increases in K1 and DSG1 and the repression of β1-integrin induced by FGF7 stimulation, while the AKT inhibitor shows an inhibitory effect only on FGF7-induced up-modulation of K1 and DSG1, and not on β1-integrin reduction. The MEK1/2 inhibitor does not display any effect on the differentiation marker modulation induced by FGF7. Equal loading was assessed with antiactin, antitubulin, and anti-GAPDH antibodies. Densitometric analysis and Student's *t* test were performed as reported in the legend to Fig. 2. *, *P* < 0.05 versus the results for the corresponding FGF7-unstimulated cells. For comparison to the results for the corresponding substrate inhibitor-untreated cells: **, *P* < 0.05; NS, not significant. (B) HaCaT pBp and HaCaT pBp-FGFR2b clones were transiently transfected with JNK1 siRNA or an unrelated siRNA as a control. Cells were then left untreated or stimulated with FGF7 as described in Materials and Methods. Western blot analysis shows that JNK1 silencing strongly damps the increases in K1 and DSG1 and rescues the repression of β1-integrin induced by FGF7 stimulation. Equal loading was assessed with antiactin antibody. Densitometric analysis and Student's *t* test were performed as reported in the legend to Fig. 2. *, *P* < 0.05 versus the results for the corresponding FGF7-unstimulated cells. For comparison to the results for the corresponding control siRNA cells: **, *P* < 0.05; NS, not significant.

Then, to assess whether JNK1 could be the main signaling pathway at the crossroad between FGFR2b-triggered autophagy and differentiation, we first analyzed the impacts of the different substrate inhibitors on the modulation of early differentiation markers induced by FGFR2b. Western blot analysis clearly showed that, particularly in HaCaT cells overexpressing FGFR2b, the JNK inhibitor was able to impair the increase in K1 and DSG1, as well as the repression of β1-integrin induced by FGF7 stimulation (Fig. 5A). Interestingly, the appreciable, even if less-evident, effect of this inhibitor also on the differentiation marker levels in cells not stimulated with FGF7 (Fig. 5A) suggested that JNK signaling controls both basal and FGF7-induced keratinocyte differentiation. On the other hand, the AKT inhibitor (Fig. 5A) displayed an inhibitory effect only on FGF7-induced up-modulation of K1 and DSG1, and not on β1-integrin reduction, suggesting that the PI3K/AKT pathway could play only a marginal role, possibly restricted to the control of some events of FGFR2b-induced early differentiation. Finally, the MEK1/2 inhibitor did not display effects on the differentiation marker modulation induced by FGF7 (Fig. 5A). The results obtained by biochemical approaches were also strengthened by those of quantitative immunofluorescence analysis, which, particularly in pBp-FGFR2b cells, showed pronounced negative effects of the JNK inhibitor on the increase in K1 and on the decrease in β1-integrin induced by FGF7 stimulation (Fig. S2). Western blot analysis performed in cells transfected with the specific siRNA showed that JNK1 silencing strongly dampened the increases in K1 and DSG1 (Fig. 5B) and rescued the repression of β1-integrin induced by FGF7 stimulation (Fig. 5B). In addition, similar to what was observed after pharmacological inhibition of JNK, JNK1 depletion also appeared to have a small effect on the basal levels of K1 and DSG1, confirming the hypothesis of a general role of this substrate in the control of keratinocyte early differentiation.

Taken together, our findings indicate that JNK1 is the exclusive FGFR2b downstream substrate, acting as a hub molecule at the crossroad between FGFR2b-triggered autophagy and differentiation. In addition, our results shed more light on the molecular mechanisms underlying these processes and clarify that the simultaneous positive regulation of autophagy and differentiation mediated by FGFR2b takes place at the shift from the basal to the suprabasal epidermal layer.

## MATERIALS AND METHODS

### Cells and treatments.

Cells from the human keratinocyte cell line HaCaT (20), stably overexpressing FGFR2b (pBp-FGFR2b) or the empty vector (pBp) and generated as previously described (21), were cultured in Dulbecco's modified Eagle's medium (DMEM) supplemented with 10% fetal bovine serum (FBS) plus antibiotics. Primary cultures of human fibroblasts derived from healthy skin (HFs) were obtained from patients attending the Dermatology Unit of the Sant'Andrea Hospital of Rome; all patients were extensively informed and their consent for the investigation was given and collected in written form in accordance with guidelines approved by the management of the Sant'Andrea Hospital. HFs were isolated and cultured as previously described (41).

Cells were transiently transfected with the pDest-mCherry-EGFP tandem expression vector containing LC3 (HaCaT mCherry-EGFP-LC3) (38), using jetPEI DNA transfection reagent (Polyplus-transfection, New York, NY) according to the manufacturer's instructions.

For RNA interference and JNK1 or ATG5 silencing, cells were transfected with JNK1 small interfering RNA (JNK1 siRNA) (Santa Cruz Biotechnology, Inc., Santa Cruz, CA), ATG5 siRNA (Santa Cruz Biotechnology), or an unrelated siRNA as a control, using Lipofectamine 2000 transfection reagent (Invitrogen, Carlsbad, CA) according to the manufacturer's protocol.

For growth factor stimulation, cells were left untreated or incubated with 100 ng/ml FGF7 (Upstate Biotechnology, Lake Placid, NY) for 24 h at 37°C.

To inhibit AKT, ERK, or JNK, cells were incubated with the AKT-specific inhibitor Akt-I-1/2, (1 μM; Calbiochem, Nottingham, UK), the MEK1/2-specific inhibitor PD0325901 (1 μM; Sigma-Aldrich, Saint Louis, MO), or the JNK-specific inhibitor SP600125 (50 μM; Sigma), respectively, for 1 h at 37°C before being treated with FGF7 in the presence of each inhibitor.

To generally inhibit autophagosome assembly, cells were treated with the class I/III PI3K/PtdIns3K inhibitor 3-methyladenine (3-MA) (5 mM, catalog number M9281; Sigma) for 1 h at 37°C before being treated with FGF7 in the presence of the inhibitor.

To inhibit keratinocyte differentiation, cells were treated with IL-22 (10 ng/ml; Peprotech, London, UK) for 1 h at 37°C before being treated with FGF7 in the presence of the inhibitor.

### Organotypic cultures.

For 3-D organotypic cultures, collagen rafts were prepared by adding 5 mg/ml rat tail type I collagen (Corning, Lowell, MA) to DMEM and reconstitution buffer (8:1:1) as previously described (42). An amount of 1 × 10^6^ HFs was added to 2 ml of the collagen mixture in polycarbonate inserts (23-mm diameter, 0.3-μm pore size; Corning) in 6-well deep-well plates (Corning). The mixture was left to polymerize for 30 min at 37°C. After 24 h, an amount of 2 × 10^5^ HaCaT pBp or pBp-FGFR2b cells was seeded on the collagen gel and left to grow for a week in complete medium added in both the top and the bottom wells. Then, the organotypic cultures were raised to the air-liquid interface and cultured for a further 2 weeks in complete medium and left untreated or stimulated with 100 ng/ml FGF7 (Upstate Biotechnology) for the last 4 days. Rafts were finally fixed in 10% formalin and embedded in paraffin, and 4-μm slices were obtained.

### Immunofluorescence.

HaCaT clones, grown on coverslips, were fixed with 4% paraformaldehyde in phosphate-buffered saline (PBS) for 30 min at 25°C, followed by treatment with 0.1 M glycine for 20 min at 25°C and with 0.1% Triton X-100 for an additional 5 min at 25°C to allow permeabilization. The cells were then incubated for 1 h at 25°C with the following primary antibodies: mouse monoclonal anti-LC3 antibody (1:100 in PBS, 5F10; Nanotools, Teningen, Germany), mouse monoclonal anti-β1-integrin antibody (1:1,000 in PBS, ST2-16; Santa Cruz Biotechnology), rabbit polyclonal anti-K1 antibody (1:50 in PBS, AF 87; Covance, Princeton, NJ), and rabbit polyclonal anti-Bek antibody (1:50 in PBS, C-17; Santa Cruz Biotechnology). The primary antibodies were visualized using goat anti-rabbit IgG–Texas red (1:200 in PBS; Jackson ImmunoResearch Laboratories, West Grove, PA) and goat anti-mouse IgG–Alexa Fluor 488 (1:200 in PBS; Life Technologies, Carlsbad, CA) for 30 min at 25°C. Nuclei were stained with DAPI (4′,6-diamidino-2-phenylindole) (1:1,000 in PBS; Sigma). Coverslips were finally mounted with Mowiol (Sigma) for observation. Organotypic raft sections, obtained as described above, were deparaffinized in xylene and rehydrated through graded ethanol dilutions to PBS, pH 7.4. Antigen retrieval was achieved by heating sections in low-pH target retrieval solution (Dako, Carpinteria, CA) for 15 min at 97°C. Sections were then washed with PBS and blocked using 10% bovine calf serum and 0.2% Triton X-100 for 30 min at 25°C before staining with rabbit polyclonal anti-K1 antibody (1:500 in PBS, AF 87; Covance) or mouse monoclonal anti-LC3 antibody (1:100 in PBS; Nanotools) for 1 h in a humidified chamber. Slides were washed extensively in PBS, and the primary antibodies were visualized using goat anti-rabbit IgG–Texas red (1:200 in PBS; Jackson ImmunoResearch Laboratories) and goat anti-mouse IgG–Alexa Fluor 488 (1:200 in PBS; Life Technologies) for 30 min at 25°C. Nuclei were stained with DAPI (1:1,000 in PBS; Sigma). Sections were finally mounted with Mowiol (Sigma).

Fluorescence signals were analyzed by conventional fluorescence or by scanning cells in a series of sequential sections with an ApoTome system (Zeiss, Oberkochen, Germany) connected with an Axiovert 200 inverted microscope (Zeiss); image analysis was performed by using the Axiovision software (Zeiss), and images were obtained by 3-D reconstruction of the total number of the serial optical sections. Quantitative analysis of the fluorescence intensity was performed by using the Axiovision software (Zeiss), analyzing 10 different fields randomly taken from 3 independent experiments. Quantitative analysis of LC3-positive dots per cell was performed by analyzing 100 cells for each sample in 5 different microscopy fields from 3 different experiments. Results are shown as mean values ± standard errors (SE). Student's *t* test was performed, with significance levels defined as *P* values of <0.05.

### Western blot analysis.

Cells were lysed in a buffer containing 50 mM HEPES, pH 7.5, 150 mM NaCl, 1% glycerol, 1% Triton X-100, 1.5 mM MgCl_2_, 5 mM EGTA, supplemented with protease inhibitors (10 μg/ml aprotinin, 1 mM phenylmethylsulfonyl fluoride [PMSF], 10 μg/ml leupeptin) and phosphatase inhibitors (1 mM sodium orthovanadate, 20 mM sodium pyrophosphate, 0.5 M NaF). A range of 20 to 50 μg of total protein was resolved under reducing conditions by 8 or 12% SDS-PAGE and transferred to reinforced nitrocellulose (BA-S 83; Schleicher & Schuell, Keene, NH). The membranes were blocked with 5% nonfat dry milk in PBS–0.1% Tween 20 and incubated with anti-E-cadherin (NCH-38; Dako), anti-DSG1 (27B2; Life Technologies), anti-β1-integrin (ST2-16; Santa Cruz Biotechnology), and anti-phospho-JNK (anti-p-JNK) (Thr183/Tyr185, 9255S; Cell Signaling Technology, Beverly, MA) monoclonal antibodies or with anti-LC3 (MBL, Woburn, MA), anti-p-p44/42 mitogen-activated protein kinase (MAPK) (p-ERK1/2) (Thr202/Tyr204; Cell Signaling), anti-K1 (AF 87; Covance), anti-p-AKT (Ser 473; Cell Signaling), anti-Bek (C-17; Santa Cruz Biotechnology), anti-p-BCL2 (Ser 70; Cell Signaling), and anti-ATG5 (Novus Biologicals, Littleton, CO) polyclonal antibodies, followed by enhanced chemiluminescence (ECL) detection (Amersham, Arlington Heights, IL). The membranes were rehydrated by being washed in PBS-Tween 20, stripped with 100 mM mercaptoethanol and 2% SDS for 30 min at 55°C, and probed again with anti-JNK (Cell Signaling), anti-AKT (H-136; Santa Cruz Biotechnology), anti-p44/42 MAPK (ERK1/2) (137F5; Cell Signaling), and anti-α-tubulin (2148S; Cell Signaling) polyclonal antibodies or with anti-BCL2 (124; Cell Signaling), anti-β-actin (AC-15; Sigma), and anti-glyceraldehyde-3-phosphate dehydrogenase (anti-GAPDH) (Santa Cruz Biotechnology) monoclonal antibodies to estimate protein equal loading. Densitometric analysis was performed using Quantity One software (Bio-Rad Laboratories, Hercules, CA). The resulting values from three different experiments were normalized, expressed as fold increases with respect to the control value, and reported as mean values ± standard deviations (SD). Student's *t* test was performed, with significance levels defined as *P* values of <0.05.

### Primers.

Oligonucleotide primers necessary for target genes and the housekeeping gene were chosen by using the online tool Primer-BLAST (43) and purchased from Invitrogen. The following primers were used: for the MAP1LC3B target gene, 5′-CGCACCTTCGAACAAAGAG-3′ (sense) and 5′-CTCACCCTTGTATCGTTCTATTATCA-3′ (antisense); for the BECN1 target gene, 5′-GGATGGTGTCTCTCGCAGAT-3′ (sense) and 5′-TTGGCACTTTCTGTGGACAT-3′ (antisense); for the ATG5 target gene, 5′-CAACTTGTTTCACGCTATATCAGG-3′ (sense) and 5′-CACTTTGTCAGTTACCAACGTCA-3′ (antisense); for the K10 target gene, 5′-GATGTGAATGTGGAAATGAATGCTG-3′ (sense) and 5′-TGTAGTCAGTTCCTTGCTCTTTTCA-3′ (antisense); for the DSG1 target gene, 5′-GTGGGAGAAAGAAAAAGAACAGAGAAG-3′ (sense) and 5′-CTACCACCACCAGAAAAATGAACAG-3′ (antisense); for the FGFR2b target gene, 5′-CGTGGAAAAGAACGGCAGTAAATA-3′ (sense) and 5′-GAACTATTTATCCCCGAGTGCTTG-3′ (antisense); and for the 18S rRNA housekeeping gene, 5′-CGAGCCGCCTGGATACC-3′ (sense) and 5′-CATGGCCTCAGTTCCGAAAA-3′ (antisense). Oligonucleotide primers for the K1 target gene were purchased from Bio-Rad. For each primer pair, we performed no-template control and no-reverse-transcriptase control (reverse transcription [RT]-negative) assays, which produced negligible signals.

### RNA extraction and cDNA synthesis.

Organotypic cultures were deparaffinized, and RNA was extracted using the TRIzol method (Invitrogen) according to the manufacturer's instructions and eluted with 0.1% diethylpyrocarbonate (DEPC)-treated water. Each sample was treated with DNase I (Invitrogen). The total RNA concentration was quantitated by spectrophotometry; 1 μg of total RNA was used for reverse transcription using the iScriptTM cDNA synthesis kit (Bio-Rad) according to the manufacturer's instructions.

### PCR amplification and real-time quantitation.

Real-time RT-PCR was performed using the iCycler real-time detection system (iQ5 Bio-Rad) with optimized PCR conditions. The reactions were carried out in a 96-well plate using iQ SYBR green supermix (Bio-Rad), adding forward and reverse primers for each gene and 1 μl of diluted template cDNA to a final reaction mixture volume of 15 μl. All assays included a negative control and were replicated three times. The thermal cycling program was performed as described previously (18). Real-time quantitation was performed with the help of the iCycler IQ optical system software, version 3.0a (Bio-Rad), according to the manufacturer's manual. Results are reported as mean values ± SE from three different experiments in triplicate. Student's *t* test was performed, with significance levels defined as *P* values of <0.05.

## Supplementary Material

Supplemental material
