# Supplementary material for: Role of Fibroblast Growth Factor Receptor 2b in the Cross Talk between Autophagy and Differentiation: Involvement of Jun N-Terminal Protein Kinase Signaling
Source: Mol Cell Biol. 2018 Jun 14;38(13):e00119-18. doi: 10.1128/MCB.00119-18 (PMC6002692; doi:10.1128/MCB.00119-18)
Supplement: Supplemental material [file supp_38_13_e00119-18__index.html]

Supplemental material 

# Role of Fibroblast Growth Factor Receptor 2b in the Cross Talk between Autophagy and Differentiation: Involvement of Jun N-Terminal Protein Kinase Signaling

## Supplemental material

- Supplemental file 1 -

  Fig. S1 (FGFR2b-induced autophagy and differentiation) and S2 (Inhibition of JNK signaling and FGFR2b-mediated differentiation)

  PDF, 2.1M
