# Supplementary material for: Role of Fibroblast Growth Factor Receptor 2b in the Cross Talk between Autophagy and Differentiation: Involvement of Jun N-Terminal Protein Kinase Signaling
Source: Mol Cell Biol. 2018 Jun 14;38(13):e00119-18. doi: 10.1128/MCB.00119-18 (PMC6002692; doi:10.1128/MCB.00119-18)
Supplement: Supplemental material [file MCB.00119-18_zmb999101789s1.pdf]

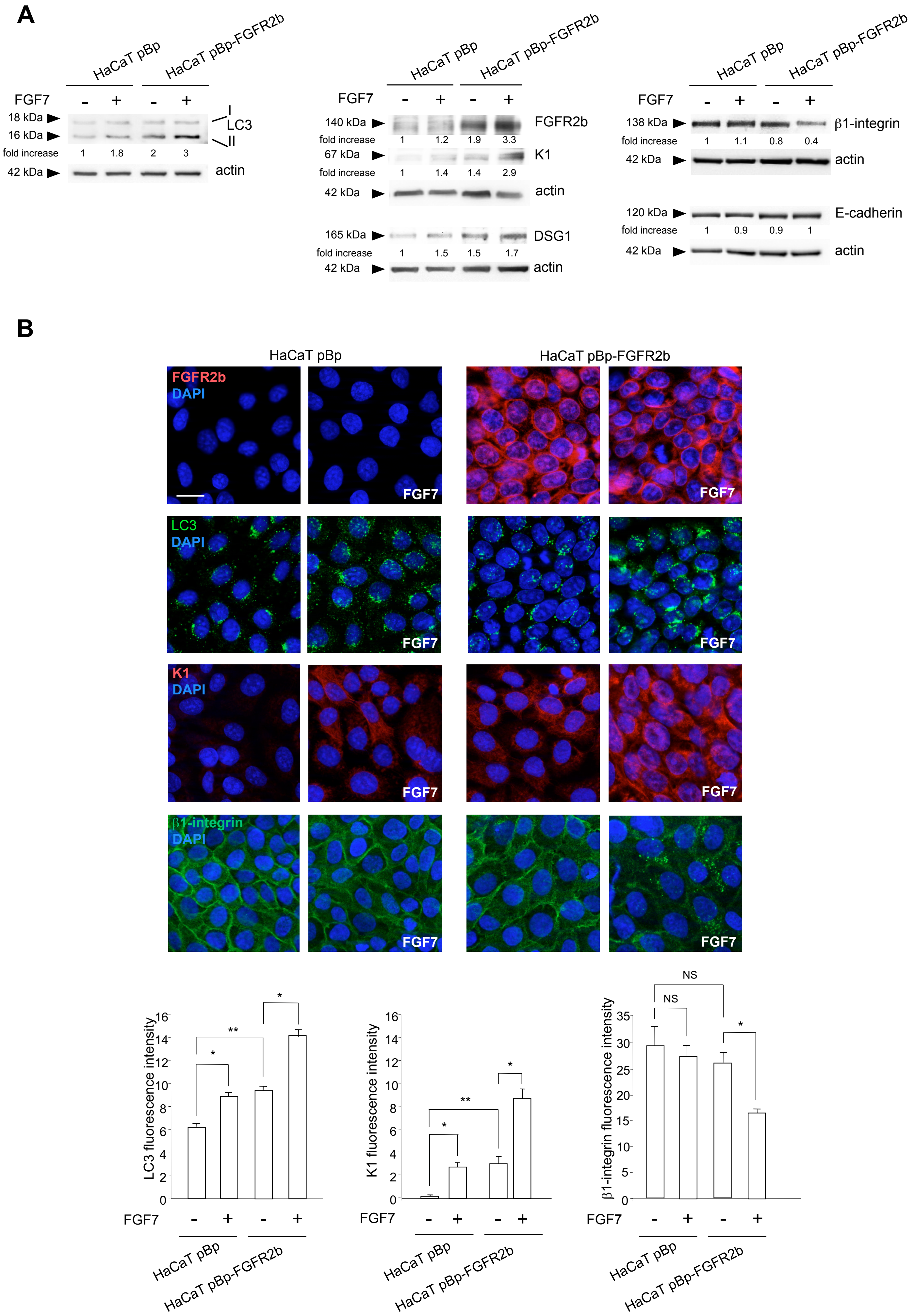

Supplementary Figure S1

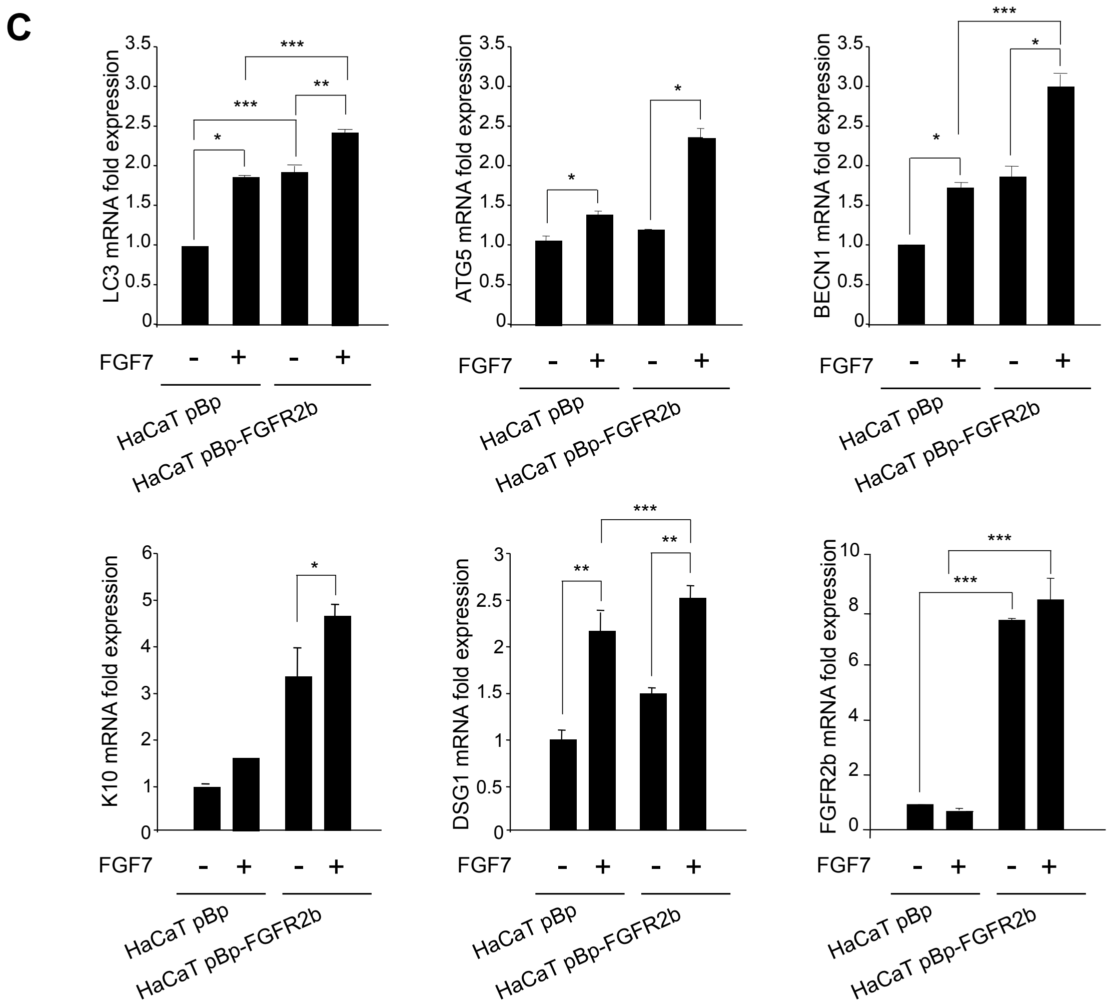

Supplementary Figure S1. FGFR2b expression and signaling induce both autophagy and early differentiation in confluent HaCaT cells. HaCaT pBp and HaCaT pBp-FGFR2b were left untreated or stimulated with FGF7 as reported in Material and Methods. (A) Western blot analysis shows that, upon FGF7 stimulation, the levels of LC3-II, K1 and DSG1 are increased in HaCaT pBp-FGFR2b cells compared to controls, particularly in response to FGF7 (left and central panels). In contrast, the level of β1-integrin decreases only in HaCaT pBp-FGFR2b cells in response to FGF7, while appears unaltered in control cells (right panel). Both clones show no changes in E-cadherin expression (right panel). The band corresponding to the molecular weight of FGFR2 is increased in HaCaT pBp-FGFR2b cells compared to HaCaT pBp cells (central panel). The equal loading was assessed with anti-actin antibody. The values of the densitometric analysis are relative to the experiment shown, which is the representative of three independent. (B) Quantitative immunofluorescence analysis confirms that LC3 and K1 signal increase in FGFR2b overexpressing cells and more evidently in response to FGF7. In contrast, β1-integrin staining is significantly decreased and delocalized from the plasma membrane to intracellular dots only in HaCaT pBp-FGFR2b clones upon FGF7 stimulation. Detectable levels of FGFR2b staining are observed only in HaCaT pBp-FGFR2b cells. Quantitative analysis of the fluorescence intensity was performed as described in Material and Methods and results are expressed as mean values ± standard errors (SE). Student's t test was performed and significance levels have been defined as $p < 0.05$: $*p < 0.001$ vs the corresponding FGF7-unstimulated cells; $**p < 0.001$ vs the corresponding pBp cells; NS (not significant) vs the corresponding FGF7-unstimulated cells and vs the corresponding pBp cells. Bar: 10 μm. (C) Real-time RT-PCR analysis shows that mRNA expression of the reported autophagic and differentiation markers are increased in pBp-FGFR2b cells particularly after the stimulation with FGF7. Higher levels of FGFR2b mRNA transcript are detected in HaCaT pBp-FGFR2b cells compared to HaCaT pBp cells. Results are expressed as mean values ± SE. Student's t test was performed and significance levels have been defined as $p < 0.05$: $*p < 0.01$ and $**p < 0.05$ vs the corresponding FGF7-unstimulated cells; $***p < 0.05$ vs the corresponding pBp-cells.

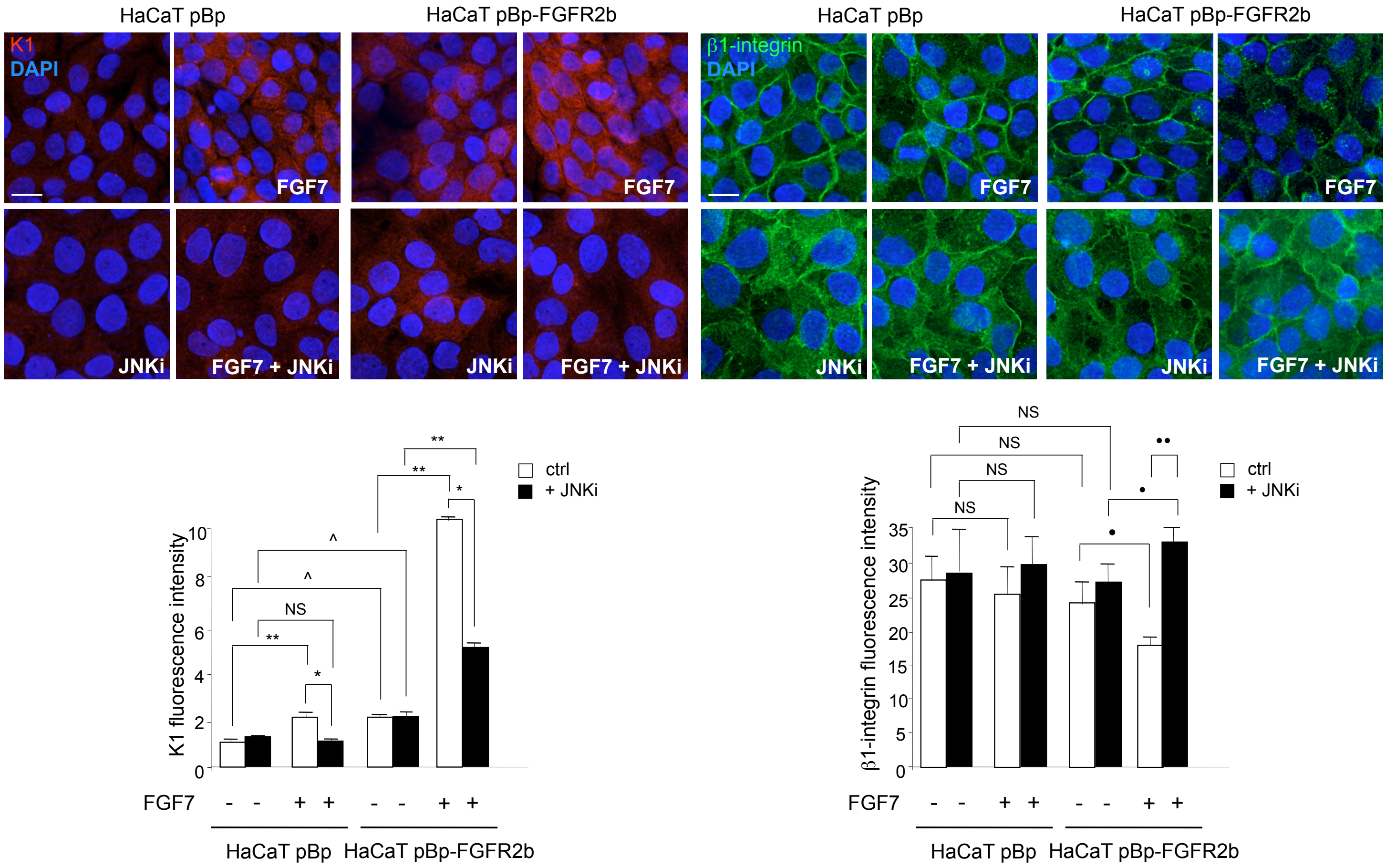

Supplementary Figure S2. The inhibition of JNK signaling blocks FGFR2b-mediated differentiation. HaCaT pBp and HaCaT pBp-FGFR2b clones were left untreated or stimulated with FGF7 in presence or not of JNK inhibitor as above. Quantitative immunofluorescence analysis confirms that JNK inhibitor interferes with the increase of K1 signal and with the repression of β1-integrin staining induced by FGF7 stimulation. The quantitative analysis and Student's t test were performed as above. NS, *p < 0.01 and ••p < 0.001 vs the corresponding control cells; NS, **p < 0.001 and •p < 0.01 vs the corresponding FGF7-unstimulated cells; ^p < 0.001 vs the corresponding pBp cells. Bar: 10 μm.
